# Clinical and structural damage outcomes in axial spondyloarthritis patients receiving NSAIDs or advanced therapies: a description of a real-life cohort

**DOI:** 10.3389/fmed.2024.1425449

**Published:** 2024-06-20

**Authors:** Anastasia Mocritcaia, Chafik Chacur, C. D. Adao Abe, Ana Belén Azuaga-Piñango, Beatriz Frade-Sosa, Juan C. Sarmiento-Monroy, Lucia Alascio, J. A. Gómez-Puerta, Raimon Sanmartí, Juan D. Cañete, Julio Ramírez

**Affiliations:** Rheumatology Department, Hospital Clínic, Barcelona, Spain

**Keywords:** axial spondyloarthritis, NSAIDs, assessment, management, biological therapy

## Abstract

**Introduction:**

This study aims to describe the clinical characteristics, disease activity, and structural damage in patients with axial spondyloarthritis (axSpA) who receive chronic treatment with nonsteroideal anti-inflammatory drugs (NSAIDs) or advanced therapies in a clinical setting.

**Methods:**

Cross-sectional study on axSpA patients consecutively recruited from the outpatient clinic of a tertiary hospital. We collected data on clinical and demographic characteristics, as well as treatment patterns involving NSAIDs and advanced therapies. Structural damage was assessed using mSASSS.

**Results:**

Overall, data from 193 axSpA patients (83% ankylosing spondylitis) were gathered, with a mean disease duration of 21.4 years. Of these, 85 patients (44%) were exclusively taking NSAIDs, while 108 (56%) were receiving advanced therapies, with TNF inhibitors being the predominant choice (93 out of 108, 86.1%). Among patients using NSAIDs, 64.7% followed an on-demand dosing regimen, while only 17.6% used full doses. Disease activity was low, with a mean BASDAI of 3.1 and a mean ASDAS-CRP of 1.8. In comparison to patients under chronic NSAID treatment, those taking advanced therapies were primarily male (69.4% versus 51.8%, *p* = 0.025) and significantly younger (mean age of 49 versus 53.9 years, *p* = 0.033). Additionally, patients on advanced therapies exhibited lower ASDAS-CRP (*p* = 0.046), although CRP serum levels and BASDAI scores did not differ between the two groups. In the multivariable analysis, therapy (NSAID versus biological treatment) was not independently associated with ASDAS-CRP, BASDAI or mSASSS.

**Conclusion:**

This cross-sectional analysis of a real-world cohort of axSpA patients shows positive clinical and radiological outcomes for both NSAIDs and advanced therapies.

## 1 Introduction

Axial spondyloarthritis (axSpA) is an inflammatory chronic disease characterized by predominant involvement of the axial skeleton, which includes two entities: ankylosing spondylitis (AS) or radiographic (r-axSpA) and non-radiographic axSpA (nr-axSpA) (1, 2). These are considered two distinct subtypes within the same disease spectrum. AS, in its most severe manifestation, is characterized by severe physical immobility and functional disability. Furthermore, patients with nr-axSpA may progress to r-axSpA. This progression to AS has been reported to occur in 5–12% of cases after 2 years and in approximately 25% of patients after 15 years (3–6).

Non-Steroidal Anti-Inflammatory Drugs (NSAIDs) and advanced therapies, including TNF, IL-17, and JAK inhibitors, are the primary pharmacological options available for treating axSpA patients. According to the Assessment of SpondyloArthritis international Society (ASAS) recommendations, treatment should be guided by a predefined target. The Ankylosing Spondylitis Disease Activity Score (ASDAS) is the most suitable instrument for this purpose, given its relation to syndesmophyte formation (7). If the target has not been achieved after a full course of NSAIDs at the maximum tolerated dose, advanced therapies such as TNF, IL-17, and JAK inhibitors are recommended.

Clear data regarding the chronic use of NSAIDs in the clinical management of axSpA, including intake patterns and dosages, and whether these patients achieve therapeutic goals is currently lacking. While some observational studies have indicated that approximately 70% of AS patients adhere to regular NSAIDs usage, limited information is available on patients who rely solely on NSAIDs as an exclusive treatment for axSpA (8).

In the double-blind, placebo controlled INFAST study, the combination therapy with infliximab plus naproxen was superior to naproxen monotherapy for reaching ASAS partial remission (61.9% versus 35.3% at week 28, *p* = 0.002) in patients with early, active axSpA who were naïve to NSAIDs or received a submaximal dose of NSAIDs. However, up to one third of naproxen patients achieved the therapy goal, suggesting a beneficial effect of NSAIDs as initial strategy in a good proportion of axSpA patients (9).

Ideally, when NSAIDs are used exclusively to manage the signs and symptoms of the disease, it is presumed that most patients achieve the therapeutic targets recommended by ASAS. This would suggest that there is no additional benefit to be gained from transitioning to advanced therapies, neither in terms of symptom relief nor prevention of structural damage. However, a recent study from a Spanish cohort has reported that disease control in AS was significantly more effective with biological therapies than with NSAIDs, particularly regarding achieving remission and low disease activity (10).

The primary objective of this study is to describe the clinical characteristics, disease activity, and structural damage in axSpA patients undergoing chronic treatment with NSAIDs or advanced therapies. Our working hypothesis is that the chronic use of both NSAIDs and advanced therapies, following the rheumatologist’s decision in clinical practice, is associated with long-term favorable clinical and radiological outcomes in axSpA patients.

## 2 Patients and methods

We conducted a cross-sectional study involving patients diagnosed with axSpA based on the ASAS criteria (11, 12). These patients were consecutively recruited from the outpatient clinic of the Rheumatology Department at the Hospital Clinic of Barcelona, Spain between January and December 2022.

A comprehensive examination of their clinical and demographic characteristics was carried out. Data included age, sex, diagnosis (r-axSpA versusnr-axSpA), disease duration, relevant comorbidities associated with spondyloarthritis (such as psoriasis and inflammatory bowel disease [IBD]), history of uveitis, enthesitis (according to the clinician’s criteria), peripheral arthritis, HLA-B27 positivity, and treatment history. Disease activity was assessed using both the Bath Ankylosing Spondylitis Disease Activity Index (BASDAI) and the Ankylosing Spondylitis Disease Activity Score based on C-reactive protein (ASDAS-CRP). Structural damage was evaluated by analyzing radiographs of patients’ cervical and lumbar spines, utilizing the modified Stoke Ankylosing Spondylitis Spine Score (mSASSS) as recommended by ASAS (13). All analyzed radiographies were taken during the 12 months before the data collection.

To ensure the consistency and reliability of radiography scoring, a small subset of the radiographs was independently scored twice by two readers (AM and CC) with a 48 to 72-h gap. The intrareader agreement was 0.86 and 0.95 for AM and CC, respectively, and the interreader agreement was 0.71.

Additionally, patients were categorized based on the treatment they received, which included NSAIDs, conventional Disease-Modifying Antirheumatic Drugs (DMARDs) like methotrexate and leflunomide (for patients with both axial and peripheral forms), and biologic DMARDs. The patterns of treatment were recorded. Patients receiving NSAIDs were further classified according to their intake frequency, which included daily full doses, reduced doses, and on-demand patterns. Patients undergoing advanced therapies were also categorized based on intake frequency, which included full doses or tapering doses. Some patients on advanced therapy were also taking NSAIDs. This data was analyzed in the general description of the cohort, but not considered when comparing NSAID monotherapy versus advanced therapy. The decision to initiate NSAIDs or advanced therapies was made by the treating clinician, and there was no specific protocol used for this study, apart from following national guidelines for the treatment of axSpA (14).

## 3 Statistical analysis

A descriptive analysis was conducted. Categorical and quantitative variables were described as frequencies, percentage and mean + standard deviation (SD) or median + range, as appropriate. A comparison between treatment groups (NSAID monotherapy and advanced therapy) was performed. The analysis involved the use of the Chi-square test for qualitative variables and either the T-test or U-test for quantitative variables, depending on their distribution. Following this initial analysis, we conducted several multivariable analyses (linear regression) with the objective of adjusting for potential confounding factors. The 3 dependent variables used for the multivariable analysis were the structural damage as evaluated by mSASSS, and the disease activity, as evaluated by both ASDAS-CRP and BASDAI. Variables that exhibited a *p*-value of less than 0.1 in the univariate analysis and those considered clinically relevant were included in each model as covariates. These covariates aimed to account for any potential confounding factors that might influence structural damage or disease activity. Therapy (NSAIDs versus advanced therapies) was one of the covariates included in all analysis in order to investigate if the treatment at the moment of the analysis was independently related to structural damage or disease activity. Statistical analysis was carried out by SPSS Statistics 20.0 program (Chicago, Illinois, United States).

## 4 Results

**Figure 1:**
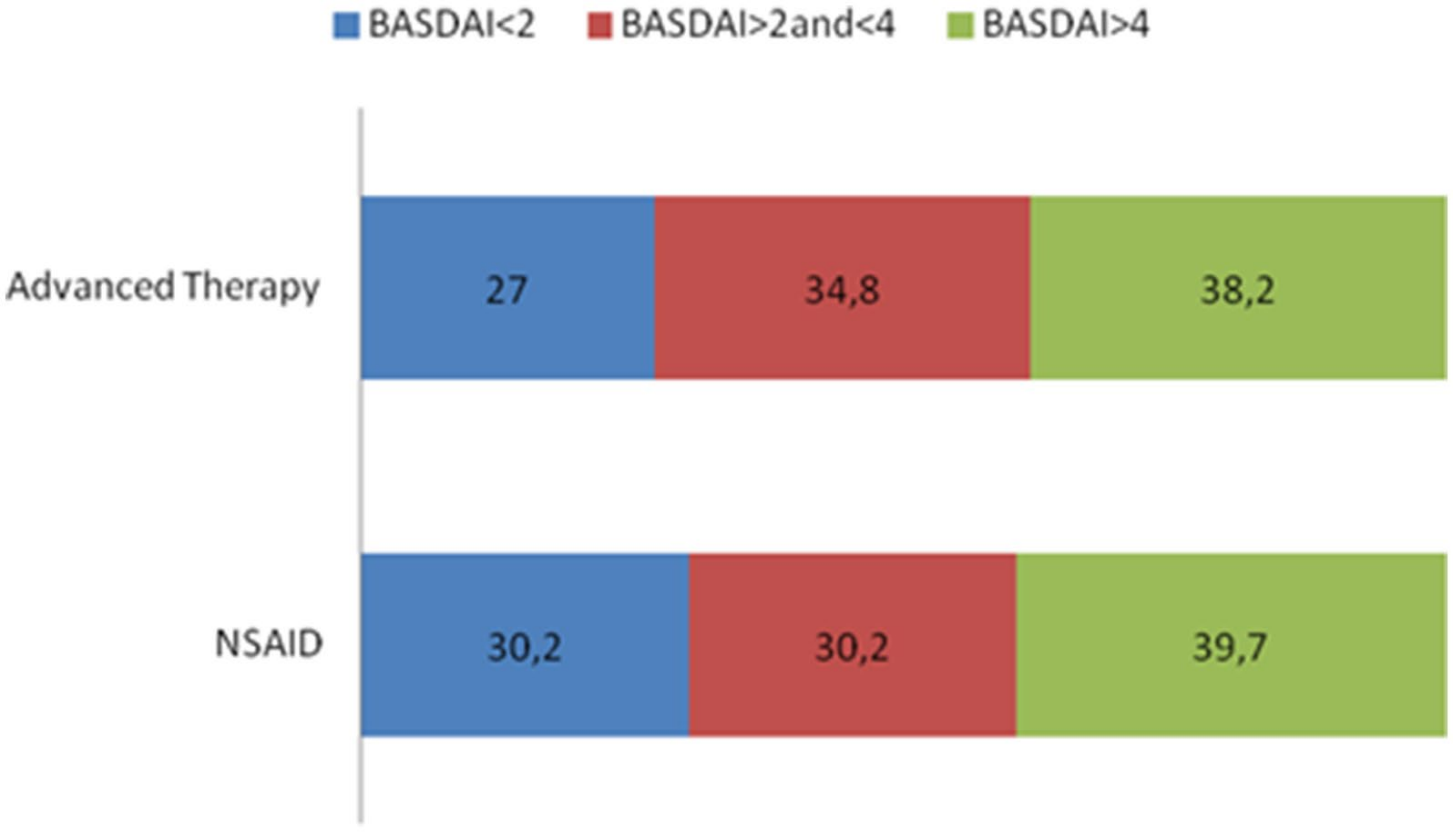
BASDAI subgroups according to disease activity.

**Figure 2:**
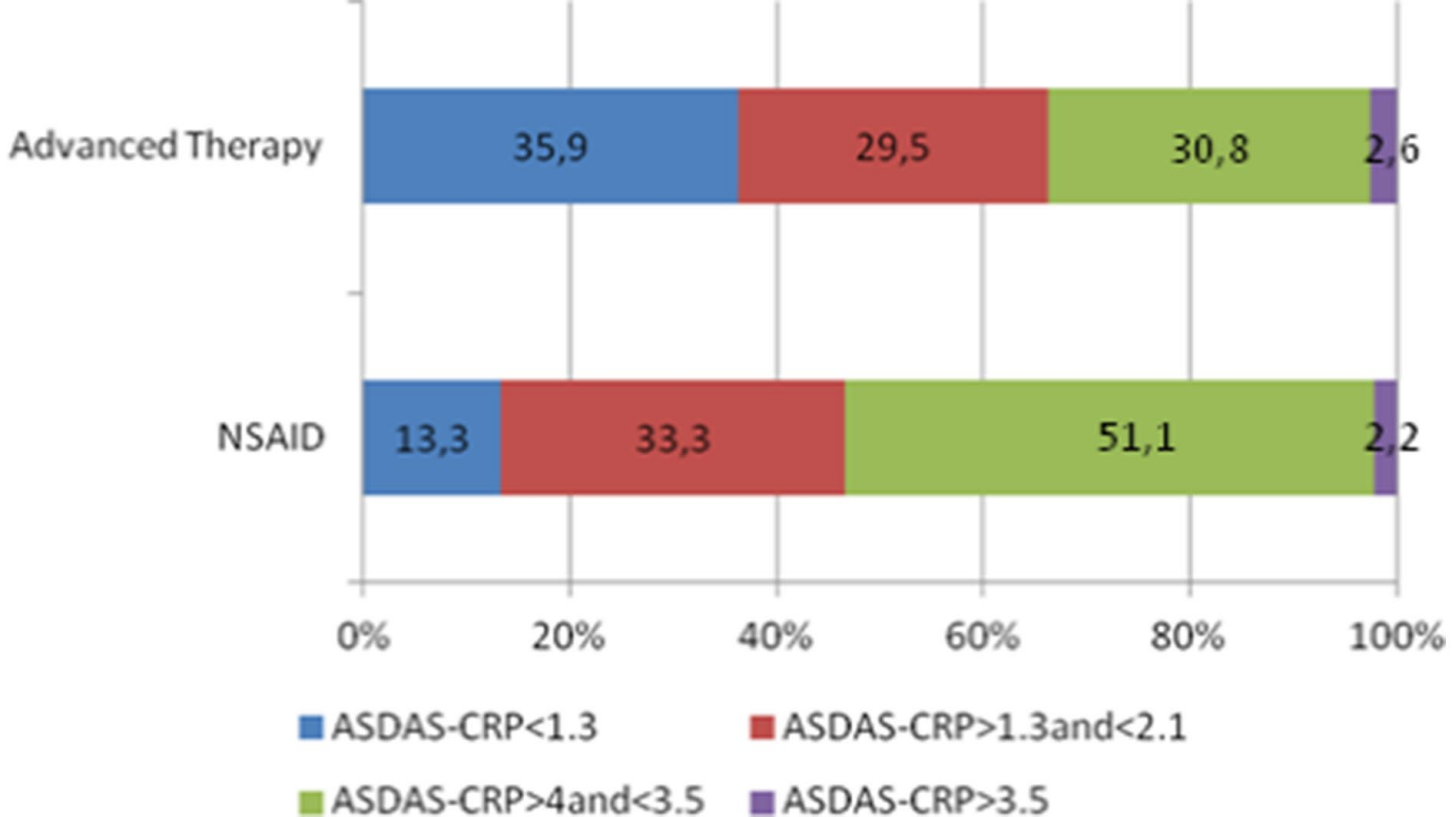
ASDAS-CRP subgroups according to disease activity.

Our study included a total of 193 patients with axSpA, predominantly diagnosed with AS. The clinical and demographic characteristics of these patients are summarized in Table 1, while details of their treatment regimens and dosages can be found in Table 2. Most of the patients were male (61.7%) with a mean age of 51 years and an average disease duration of 21.4 years. A subset of the patients presented with enthesitis (9.3%), and a considerable proportion had peripheral arthritis (26.4%). The majority were HLA-B27 positive (84.0%). Comorbid conditions included psoriasis (8.3%), IBD (3.1%), and a history of uveitis (23.8%). Disease activity was primarily low, with a mean BASDAI of 3.1 and a mean ASDAS-CRP of 1.8. Serum CRP levels averaged 0.6 mg/dL. Structural damage, as assessed by mSASSS, had a median score of 4.0. A total of 28.3% of patients achieved BASDAI remission (<2), and 61.2% attained low disease activity (<4) (Figure 1). For ASDAS-CRP, 27.6% achieved inactive disease (<1.3), and 58.5% achieved low disease activity (<2.1) (Figure 2).

**Table 1:** Demographic and clinical characteristics of axial spondyloarthritis patients taking NSAIDs and advanced therapies.

	All	NSAID group	Advanced therapy group	*p*
*n*	193	85	108	
Age (mean years ± SD)	51 ± 14.8	53.6 ± 16.4	49.0 ± 13.1	0.033
Male, *n* (%)	119 (61.7%)	44 (51.8%)	75 (69.4%)	0.025
r-axSpA (%)	160 (82.9%)	64 (74.1%)	96 (90.0%)	0.012
nr-axSpA (%)	33 (17.1%)	21 (25.9%)	12 (11.1%)	
Disease duration, mean (years ± SD)	21.4 ± 14.2	20.7 ± 15.8	22 ± 13.0	0.582
Psoriasis, *n* (%)	16 (8.3%)	6 (7.1%)	10 (9.3%)	0.793
Inflammatory bowel disease, *n* (%)	6 (3.1%)	1 (1.1%)	5 (4.6%)	0.235
Uveitis, *n* (%)	46 (23.8%)	20 (23.5%)	26 (24.1%)	1
Enthesitis, *n* (%)	18 (9.3%)	10 (11.8%)	8 (7.4%)	0.323
Peripheral arthritis, *n* (%)	51 (26.4%)	19 (23.5%)	32 (29.6%)	0.326
HLA-B27, *n* (%)	162 (84.0%)	70 (85.4%)	92 (87.7%)	1
BASDAI, mean, (U ± SD)	3.1 ± 2.1	3.2 ± 2.2	3.0 ± 2.1	0.669
CRP, mean (mg/dL ± SD)	0.6 ± 0.6	0.7 ± 0.64	0.6 ± 0.6	0.182
ASDAS-CRP, mean (U ± SD)	1.8 ± 0.9	2.1 ± 0.9	1.7 ± 0.9	0.047
MSASSS, median (U ± range)	4.0 ± 72	4.0 ± 29	4.0 ± 72	0.585

NSAID, Non-Steroidal Anti-Inflammatory Drugs; r-axSpA, radiographic axial spondyloarthritis; nr-axSpA, non-radiographic axial spondyloarthritis; HLA-B27, Human Leukocyte Antigen-B27; BASDAI, Bath Ankylosing Spondylitis Disease Activity Index; CRP, C-Reactive Protein; ASDAS-CRP, Ankylosing Spondylitis Disease Activity Score-CRP; mSASSS, modified Stoke Ankylosing Spondylitis Spinal Score.

**Table 2:** Types of treatment and dose among patients with axial spondyloarthritis taking NSAIDs and advanced therapies.

	NSAID group	Advanced therapy group
NSAIDs, *n* (%)	85 (100.0%)	22 (20.4%)
Daily full doses, *n* (%)	15 (17.6%)	2 (1.8%)
75% of full doses, *n* (%)	3 (3.5%)	0 (0.0%)
50% of full doses, *n* (%)	12 (14.1%)	0 (0.0%)
On-demand, *n* (%)	55 (64.7%)	20 (18.5%)
TNF inhibitors, *n* (%)		93 (86.1%)
Etanercept, *n* (%)		21 (19.4%)
Adalimumab, *n* (%)		41 (38.0%)
Infliximab, *n* (%)		9 (8.3%)
Golimumab, *n* (%)		19 (17.6%)
Certolizumab, *n* (%)		3 (2.8%)
IL-17 inhibitors, *n* (%)		15 (13.9%)
Secukinumab, *n* (%)		12 (11.1%)
Ixekizumab, *n* (%)		3 (2.8%)
Tapering doses, *n* (%)		35 (32.4%)
Time of evolution of advanced therapy, mean (years ± SD)		5.4 ± 4.8
Conventional DMARDs, *n* (%)	1 (1.2%)	5 (4.6%)
Methotrexate, *n* (%)	1 (1.2%)	4 (3.7%)
Leflunomide, *n* (%)	0 (0.0%)	1 (0.09%)

NSAID, Non-Steroidal Anti-Inflammatory Drugs; DMARDs, Disease-Modifying Antirheumatic Drugs.

Of the patient cohort, 44% were taking exclusively NSAIDs, and 56% were on advanced therapies, primarily TNF inhibitors (86.1%). A small subset (3.1%) received conventional synthetic DMARDs due to additional peripheral symptoms, mainly methotrexate (5 out of 6). Among patients using NSAIDs monotherapy, 64.7% followed an on-demand dosing regimen, and only 17.6% used daily full doses. Patients receiving advanced therapies were more likely to be diagnosed with AS (*p* = 0.012), were predominantly male (*p* = 0.025), and notably younger (*p* = 0.033) compared to those on NSAIDs monotherapy. The mean duration since the initiation of advanced therapy was 5.4 years. Out of the 108 patients on advanced therapies, 35 (32.4%) were taking tapered doses: Ten out of 35 were receiving etanercept 50 mg sc/10 days, 21 patients adalimumab 40 mg sc/3 weeks and 4 patients infliximab 5 mg/kg/10 weeks. In this group of patients on advanced therapy, 20.4% were concurrently taking NSAIDs, with the majority (20 out of 22) using NSAIDs on-demand.

The univariate analysis revealed no significant differences between the NSAID monotherapy and advanced therapy groups concerning disease duration, HLA-B27 positivity, enthesitis, peripheral arthritis, and the presence of extraarticular manifestations such as psoriasis and uveitis. It is worth noting that patients with comorbid psoriasis exhibited significantly higher ASDAS-CRP levels (*p* = 0.028) and serum CRP levels (*p* = 0.019). Similarly, patients with enthesitis had significantly higher ASDAS-CRP scores (*p* = 0.003), although the use of advanced therapies in this subgroup was not notably higher (*p* = 0.7). Notably, among the 6 patients diagnosed with concomitant IBD, 5 were receiving biological therapy (golimumab and adalimumab).

Disease activity and systemic inflammation, as indicated by BASDAI and CRP serum levels, did not exhibit significant differences between the NSAID and advanced therapy groups. However, patients undergoing advanced treatment demonstrated lower disease activity, particularly with respect to ASDAS-CRP (*p* = 0.046), primarily due to a higher proportion of patients achieving ASDAS-CRP inactive disease (35.9% in patients with advanced therapies versus 13.3% in patients on NSAIDs, *p* = 0.021, Figure 2). MSASSS values were numerically higher in patients on advanced therapy, but no significant differences emerged in the univariate analysis (*p* = 0.585).

After that, we conducted several multivariable analyses to investigate the relevance of the therapy as a covariate in clinical (ASDAS-CRP and BASDAI) and structural (mSASSS) outcomes. Firstly, we performed a linear regression with mSASSS as dependent variable and age, sex, disease duration, subtype of axSpA, ASDAS-CRP and therapy (NSAID or Advanced therapy) as covariates. Age (β 0.422, confidence interval [CI] 95% 0.159–0.894, *p* = 0.006) was the only factor independently related to mSASSS. In the second linear regression analysis, taking BASDAI as dependent variable and age, sex, disease duration, subtype of axSpA, mSASSS and therapy (NSAID or Advanced therapy) as covariates, no factors were found as independently related to BASDAI. Finally, taking ASDAS-CRP as dependent variable and age, sex, disease duration, subtype of axSpA, mSASSS and therapy (NSAID or advanced therapy) as covariates, age (β 0.421 CI 95% 0.005–0.057, *p* = 0.020) and disease duration (β −0.315 CI 95% -0.43-0.0001, *p* = 0.049) were significantly related to ASDAS-CRP. Therapy (NSAID or advanced therapy) was not independently related (at the moment of the analysis) to neither mSASSS (*p* = 0.083), BASDAI (*p* = 0.836) nor ASDAS-CRP (*p* = 0.505).

We conducted further analysis within the subgroup of 15 patients who required full doses of NSAIDs. This subgroup was notably younger (44.2 versus 55.5 years old, *p* = 0.006) and had a shorter disease duration (12.5 versus 22.7 years, *p* = 0.021) compared to other NSAID patients. All disease activity parameters were higher in this subgroup (mean BASDAI 4.6 versus 2.8, *p* = 0.049; mean ASDAS-CRP 2.4 versus 1.9, *p* = 0.171). Structural damage, as evaluated by mSASSS, was also higher in patients requiring full doses of NSAIDs (median mSASSS 8.5 [range 29] versus 4 [range 26]), although these comparisons were exploratory due to the small number of patients in this subgroup.

## 5 Discussion

The cornerstones of axSpA treatment encompass both non-pharmacological interventions, such as education and physical exercise, and pharmacological therapies. The overarching objectives of treatment involve symptom and inflammation control, prevention of structural damage, and mitigating long-term complications (15). Patients should also strive to maintain physical functionality and participation in social and occupational activities.

Our study provides valuable insights into real-world clinical practice, revealing that most axSpA patients achieve favorable clinical and structural outcomes, regardless of the therapy. Clinical decision of the treating rheumatologist of maintaining chronic therapy with NSAIDs or advanced therapy leads to good control of the disease. Interestingly, nearly half of axSpA patients receive chronic NSAID treatment, with two thirds of them on an on-demand basis. Patients for whom clinicians decided to continue NSAID therapy have achieved general treatment goals by effectively controlling disease signs and symptoms and averting structural damage.

According to ASAS recommendations, NSAIDs are recommended as the first-line therapy for patients experiencing pain and stiffness, up to the maximum dose, while considering the risks and benefits (7). However, the proportion of axSpA patients who maintain NSAIDs as the primary therapy to manage disease activity remains underexplored. A retrospective study in North America reported that 12 and 18% of AS and nr-axSpA patients, respectively, were treated with NSAIDs as monotherapy, but details about drug intake patterns (continuous versus on-demand) were not provided (15). In our cohort, 44% of patients were managed with NSAIDs without the need for advanced therapies. Interestingly, only 17.6% of them used daily full doses, with the majority (64.7%) relying on NSAIDs as on-demand therapy. Another Spanish cohort study, limited to AS patients, produced similar results, with 58.6% of AS patients using NSAIDs as the primary therapy, half of whom relied on an on-demand regimen, and only 25% on full doses (10).

Several concerns arise from the regular use of NSAIDs in axSpA. First, the effectiveness of NSAIDs is traditionally considered lower than that of biological therapy. However, no formal comparison has been made. A recent Chinese study in AS patients investigated the clinical efficacy of celecoxib and etanercept alone versus combined treatment. After 52 weeks, the combined group showed superior ASAS20 response rates (84%) compared to the celecoxib (44%) and etanercept (58%) groups. Structural damage assessed by MRI also favored the combined and etanercept groups over the celecoxib group. While it was an open trial, this study demonstrated, for the first time, superior efficacy in clinical and radiological outcomes of biological therapy compared to NSAIDs (16). In line with this, the Groningen Leeuwarden Ankylosing Spondylitis (GLAS) cohort, after a 52-week follow-up period, found a correlation between higher NSAID intake and increased ASDAS, regardless of the use of TNF-inhibitors (17). Furthermore, a separate study reported that NSAID intake was independently related to lower odds of achieving therapeutic goals as indicated by BASDAI remission criteria (OR 0.18), ASDAS inactive disease (OR 0.08), and Routine Assessment of Patient Index Data 3 (RAPID3) remission (OR 0.26) (18). These observational studies are potentially susceptible to biases, such as confounding by disease severity. Patients who receive regular NSAIDs in addition to biological therapy are likely to be more severely affected by the disease. On the other hand, NSAIDs have demonstrated substantial symptom relief in 60–80% of patients (19). In a recent meta-analysis of randomized controlled trials in AS, the majority of available NSAIDs significantly reduced total pain scores compared to a placebo for up to 12 weeks (20). Additionally, NSAIDs have been shown to reduce acute-phase reactant levels in the blood of AS patients (21) and decrease bone marrow edema signal intensity in the sacroiliac (SI) joints on MRI in newly diagnosed axSpA patients (22). Our data indicates that most patients taking NSAIDs exhibited effective disease control, with 60.4 and 46.6% achieving BASDAI <4 and ASDAS-CRP <2.1, respectively. Moreover, no significant differences were observed between patients receiving NSAIDs and those on advanced therapies in terms of CRP serum levels and BASDAI scores. While patients on advanced therapy exhibited significantly lower ASDAS-CRP levels, the significance disappeared in the multivariate analysis. Thus, using NSAIDs as a regular treatment strategy for axSpA did not seem to result in undertreatment.

A second concern is the ability of NSAIDs to mitigate the progression of structural damage, a matter that remains inconclusive in the literature. The German Spondyloarthritis Inception Cohort (GEPSIC) study reported significantly lower odds of mSASSS increase over a 2-year period (OR = 0.15, 95% CI 0.02–0.96) in patients with high NSAID intake (23), suggesting a potential protective effect of NSAIDs on radiographic progression in AS patients. Two separate randomized trials, one involving the COX-2 selective NSAID celecoxib and the other non-selective NSAID diclofenac, investigated the impact of NSAIDs on radiographic progression. In the celecoxib trial, TNF-inhibitor-naïve AS patients were randomized to continuous versus on-demand use. After 2 years, patients in the continuous use group experienced less radiographic progression compared to those on on-demand therapy (*p* = 0.002) (24). The diclofenac trial employed a similar design, although, in contrast to the findings in the celecoxib study, the continuous group showed slightly higher numerical progression over 2 years (*p* = 0.39) (25). These results may imply that the disease-modifying effect is predominantly associated with COX-2 selective NSAIDs, although this assumption remains inconclusive. In our cohort, where more than five different NSAIDs were utilized, no substantial differences between them were apparent, although limited subgroup sizes hindered an in-depth analysis. In our study, there was no significant difference in disease activity, measured by BASDAI, and structural damage assessed using mSASSS, between patients on NSAID treatment and those on advanced therapies. It is important to note that our results should not be interpreted as strong evidence for an equivalent effect on disease activity and structural damage between NSAIDs and advanced therapies as no longitudinal follow-up was available. Rather, they suggest the presence of an indication bias, where patients with worse prognosis factors and a higher likelihood of radiographic progression may initiate advanced therapy at an earlier stage of their disease. We believe that, in clinical practice, achieving normalized CRP levels and effective control of pain and stiffness, irrespective of the therapy used, are indicative of effective management of structural damage. Consequently, our findings align with previous studies, which have indicated good disease control in patients treated with NSAIDs (26). Interestingly, patients requiring full doses of NSAIDs in our study were generally younger, with shorter disease duration, and displayed higher disease activity and more structural damage than the rest of the NSAID group. This likely reflects a severity bias, as patients requiring higher NSAID doses initially sought symptomatic relief as their first line of therapy. Although prospective data were not analyzed, it is plausible that patients requiring full NSAID doses may eventually transition to advanced therapies. In general, in clinical practice, high NSAID doses are typically reserved for short-term use to induce remission, with a subsequent reduction in NSAID dosage once disease activity is controlled.

A third consideration is the potential association of chronic NSAID use with adverse events, which may influence the shift towards long-term biological and JAK inhibitor therapies. NSAIDs are known to be associated with various well-documented adverse effects. Special care should be exercised when treating patients with a history of arterial hypertension, gastric ulcers, IBD, renal insufficiency, or prior cardiovascular events, among other conditions (27). However, the risk of these adverse events is dose dependent. In our study, two-thirds of NSAID users employed an on-demand therapy approach, with only 17.6% requiring daily full doses. This on-demand strategy has been shown to be as effective in terms of clinical and radiographic outcomes but with fewer adverse events (25). Therefore, on-demand treatment during clinical flares is preferable to continuous daily full-dose NSAID use. Additionally, certain observational studies have demonstrated a reduced cardiovascular risk in patients with r-axSpA treated with NSAIDs in comparison to those not using NSAIDs (28, 29), likely due to the reduction in disease activity and systemic inflammation.

Despite these findings, it is essential to acknowledge some limitations of this study. Firstly, as a descriptive analysis of a real-life cohort, the results presented here cannot establish causation. Secondly, this study lacked a formal protocol, and the decision to initiate advanced therapy or continue with NSAIDs was made by clinicians. Similarly, an indication bias is likely at play, where patients continuing with NSAIDs are probably less severe cases than those necessitating advanced therapy. Therefore, the results of this study should not be interpreted as evidence of similar efficacy between NSAIDs and advanced therapy. Thirdly, the cross-sectional nature of the study meant that follow-up information and longitudinal data on mSASSS, BASDAI, CRP, and ASDAS-CRP were unavailable. Finally, our study population was predominantly male and HLA-B27 positive, which may not fully represent the diversity seen in clinical practice, especially in populations with non-radiographic axSpA. Consequently, all the results presented must be interpreted with caution, and further research is needed to address unanswered questions, such as the future role of NSAIDs in treatment protocols in axSpA or the implications between using continuous versus on-demand intake of NSAIDs in clinical practice, especially regarding structural damage.

## 6 Conclusion

Our results suggest that good clinical and radiological outcomes can be achieved with both NSAID and advanced therapies in axSpA patients in a real-world setting. The final decision of the rheumatologist in maintaining chronic therapy with either NSAID or advanced therapy is the essential point to achieve our goals. Therefore, NSAID administration represents an initial and reliable step in the treatment of axSpA in a clinical setting. The fact that most of these patients achieved satisfactory outcomes in terms of clinical and radiographic results indicates that this approach is not associated with undertreatment. Furthermore, as most of our patients exclusively used on-demand treatment during clinical flares, continuous administration of NSAIDs was not necessary to achieve therapy goals, which could potentially reduce the risk of long-term adverse effects associated with NSAID use.

## Data availability statement

The raw data supporting the conclusions of this article will be made available by the authors, without undue reservation.

## Ethics statement

Ethical approval was not required for the studies involving humans because this was a retrospective revision of patients' clinical data. The studies were conducted in accordance with the local legislation and institutional requirements. Written informed consent for participation was not required from the participants or the participants' legal guardians/next of kin in accordance with the national legislation and institutional requirements because this was a retrospective revision of patients' clinical data.

## Author contributions

AM: Investigation, Visualization, Writing – original draft. CC: Investigation, Writing – review & editing. CA: Investigation, Writing – review & editing. ABA-P: Resources, Writing – review & editing. BF-S: Resources, Writing – review & editing. JS-M: Resources, Writing – review & editing. LA: Resources, Writing – review & editing. JG-P: Methodology, Resources, Writing – review & editing. RS: Resources, Writing – review & editing. JC: Resources, Writing – review & editing. JR: Conceptualization, Formal analysis, Investigation, Methodology, Project administration, Resources, Supervision, Validation, Visualization, Writing – original draft, Writing – review & editing.

## References

[1] Deodhar A Strand V Kay J Braun J. The term “non-radiographic axial spondyloarthritis” is much more important to classify than to diagnose patients with axial spondyloarthritis. Ann Rheum Dis. (2016) 75:791–4. doi: 10.1136/annrheumdis-2015-208852, PMID: 26768406

[2] Baraliakos X Braun J. Non-radiographic axial spondyloarthritis and ankylosing spondylitis: what are the similarities and differences? RMD Open. (2015) 1:e000053. doi: 10.1136/rmdopen-2015-000053, PMID: 26557375 PMC4632143

[3] Sieper J van der Heijde D. Review: nonradiographic axial spondyloarthritis: new definition of an old disease? Arthritis Rheum. (2013) 65:543–51. doi: 10.1002/art.37803, PMID: 23233285

[4] Dougados M Demattei C van den Berg R Vo Hoang V Thevenin F Reijnierse M. Rate and predisposing factors for sacroiliac joint radiographic progression after a two-year follow-up period in recent-onset Spondyloarthritis. Arthritis Rheumatol. (2016) 68:1904–13. doi: 10.1002/art.39666, PMID: 26990518 PMC5129505

[5] Poddubnyy D Rudwaleit M Haibel H Listing J Märker-Hermann E Zeidler H. Rates and predictors of radiographic sacroiliitis progression over 2 years in patients with axial spondyloarthritis. Ann Rheum Dis. (2011) 70:1369–74. doi: 10.1136/ard.2010.145995, PMID: 21622969

[6] Wang R Gabriel SE Ward MM. Progression of nonradiographic axial Spondyloarthritis to ankylosing spondylitis: a population-based cohort study. Arthritis Rheumatol. (2016) 68:1415–21. doi: 10.1002/art.39542, PMID: 26663907 PMC5025639

[7] Ramiro S Nikiphorou E Sepriano A Ortolan A Webers C Baraliakos X. ASAS-EULAR recommendations for the management of axial spondyloarthritis: 2022 update. Ann Rheum Dis. (2023) 82:19–34. doi: 10.1136/ard-2022-223296, PMID: 36270658

[8] Dau JD Lee M Ward MM Gensler LS Brown MA Learch TJ. Opioid analgesic use in patients with ankylosing spondylitis: an analysis of the prospective study of outcomes in an ankylosing spondylitis cohort. J Rheumatol. (2018) 45:188–94. doi: 10.3899/jrheum.170630, PMID: 29196383 PMC5805598

[9] Sieper J Lenaerts J Wollenhaupt J Rudwaleit M Mazurov VI Myasoutova L. Efficacy and safety of infliximab plus naproxen versus naproxen alone in patients with early, active axial spondyloarthritis: results from the double-blind, placebo-controlled INFAST study, part 1. Ann Rheum Dis. (2014) 73:101–7. doi: 10.1136/annrheumdis-2012-203201, PMID: 23696633 PMC3888606

[10] Moreno M Arévalo M Zamora M Pontes C Oliva JC Gratacós J. Comparison of disease activity in patients with ankylosing spondylitis under TNFi or NSAID treatment, is there any difference? An observational study. Reumatol Clin. (2021) 17:192–6. doi: 10.1016/j.reuma.2019.07.005, PMID: 31558361

[11] Rudwaleit M Landewé R van der Heijde D Listing J Brandt J Braun J. The development of assessment of SpondyloArthritis international society classification criteria for axial spondyloarthritis (part I): classification of paper patients by expert opinion including uncertainty appraisal. Ann Rheum Dis. (2009) 68:770–6. doi: 10.1136/ard.2009.108217, PMID: 19297345

[12] Rudwaleit M van der Heijde D Landewé R Listing J Akkoc N Brandt J. The development of assessment of SpondyloArthritis international society classification criteria for axial spondyloarthritis (part II): validation and final selection. Ann Rheum Dis. (2009) 68:777–83. doi: 10.1136/ard.2009.108233, PMID: 19297344

[13] Creemers MCW Franssen MJAM van't Hof M Gribnau FW van de Putte L van Riel P. Assessment of outcome in ankylosing spondylitis: an extended radiographic scoring system. Ann Rheum Dis. (2005) 64:127–9. doi: 10.1136/ard.2004.020503, PMID: 15051621 PMC1755183

[14] Gratacós J del Campo D Fontecha P Fernández-Carballido C Juanola Roura X Linares Ferrando LF. Recomendaciones de la Sociedad Española de Reumatología sobre el uso de terapias biológicas en espondiloartritis axial. Reumatol Clin. (2018) 14:320–33. doi: 10.1016/j.reuma.2017.08.008, PMID: 29050839

[15] Danve A Deodhar A. Treatment of axial spondyloarthritis: an update. Nat Rev Rheumatol. (2022) 18:205–16. doi: 10.1038/s41584-022-00761-z 35273385

[16] Tu L Zhao M Wang X Kong Q Chen Z Wei Q. Etanercept/celecoxib on improving MRI inflammation of active ankylosing spondylitis: a multicenter, open-label, randomized clinical trial. Front Immunol. (2022) 13:967658. doi: 10.3389/fimmu.2022.967658 36091030 PMC9458864

[17] Carbo MJG Spoorenberg A Maas F Brouwer E Bos R Bootsma H. Ankylosing spondylitis disease activity score is related to NSAID use, especially in patients treated with TNF-α inhibitors. PLoS One. (2018) 13:e0196281. doi: 10.1371/journal.pone.0196281, PMID: 29689112 PMC5915774

[18] García-Valle A Andrés-de Llano JM Fariña-González AJ González-Benítez RD Queiro-Silva R. Construct validity of the routine assessment of patient index data 3 (RAPID3) in the evaluation of axial Spondyloarthritis. J Rheumatol. (2022) 49:36–43. doi: 10.3899/jrheum.201362, PMID: 34266987

[19] Poddubnyy D van der Heijde D. Therapeutic controversies in spondyloarthritis: nonsteroidal anti-inflammatory drugs. Rheum Dis Clin N Am. (2012) 38:601–11. doi: 10.1016/j.rdc.2012.08.005 23083758

[20] Wang R Dasgupta A Ward MM. Comparative efficacy of non-steroidal anti-inflammatory drugs in ankylosing spondylitis: a Bayesian network meta-analysis of clinical trials. Ann Rheum Dis. (2016) 75:1152–60. doi: 10.1136/annrheumdis-2015-207677, PMID: 26248636 PMC11034804

[21] Benhamou M Gossec L Dougados M. Clinical relevance of C-reactive protein in ankylosing spondylitis and evaluation of the NSAIDs/coxibs’ treatment effect on C-reactive protein. Rheumatology (Oxford). (2010) 49:536–41. doi: 10.1093/rheumatology/kep393, PMID: 20028728

[22] Varkas G Jans L Cypers H Van Praet L Carron P Elewaut D. Brief report: six-week treatment of axial Spondyloarthritis patients with an optimal dose of nonsteroidal Antiinflammatory drugs: early response to treatment in signal intensity on magnetic resonance imaging of the sacroiliac joints. Arthritis Rheumatol. (2016) 68:672–8. doi: 10.1002/art.39474, PMID: 26473982

[23] Poddubnyy D Rudwaleit M Haibel H Listing J Märker-Hermann E Zeidler H. Effect of non-steroidal anti-inflammatory drugs on radiographic spinal progression in patients with axial spondyloarthritis: results from the German Spondyloarthritis inception cohort. Ann Rheum Dis. (2012) 71:1616–22. doi: 10.1136/annrheumdis-2011-201252, PMID: 22459541

[24] Wanders A Heijde D v d Landewé R Béhier JM Calin A Olivieri I. Nonsteroidal antiinflammatory drugs reduce radiographic progression in patients with ankylosing spondylitis: a randomized clinical trial. Arthritis Rheum. (2005) 52:1756–65. doi: 10.1002/art.21054, PMID: 15934081

[25] Sieper J Listing J Poddubnyy D Song IH Hermann KG Callhoff J. Effect of continuous versus on-demand treatment of ankylosing spondylitis with diclofenac over 2 years on radiographic progression of the spine: results from a randomised multicentre trial (ENRADAS). Ann Rheum Dis. (2016) 75:1438–43. doi: 10.1136/annrheumdis-2015-207897, PMID: 26242443

[26] Ortolan A Webers C Sepriano A Falzon L Baraliakos X Landewé RB. Efficacy and safety of non-pharmacological and non-biological interventions: a systematic literature review informing the 2022 update of the ASAS/EULAR recommendations for the management of axial spondyloarthritis. Ann Rheum Dis. (2023) 82:142–52. doi: 10.1136/ard-2022-223297, PMID: 36261247

[27] Bindu S Mazumder S Bandyopadhyay U. Non-steroidal anti-inflammatory drugs (NSAIDs) and organ damage: a current perspective. Biochem Pharmacol. (2020) 180:114147. doi: 10.1016/j.bcp.2020.114147, PMID: 32653589 PMC7347500

[28] Tam HW Yeo KJ Leong PY Chen CH Li YC Ma CM. Sulfasalazine might reduce risk of cardiovascular diseases in patients with ankylosing spondylitis: a nationwide population-based retrospective cohort study. Int J Rheum Dis. (2017) 20:363–70. doi: 10.1111/1756-185X.12986, PMID: 27943609

[29] Wu LC Leong PY Yeo KJ Li TY Wang YH Chiou JY. Celecoxib and sulfasalazine had negative association with coronary artery diseases in patients with ankylosing spondylitis: a nation-wide, population-based case-control study. Medicine. (2016) 95:e4792. doi: 10.1097/MD.0000000000004792, PMID: 27603385 PMC5023908

